# The Effect of Acetyl Salicylic Acid Induced Nitric Oxide Synthesis in the Normalization of Hypertension through the Stimulation of Renal Cortexin Synthesis and by the Inhibition of Dermcidin Isoform 2, A Hypertensive Protein Production

**Published:** 2014-09

**Authors:** Rajeshwary Ghosh, Sarbashri Bank, Uttam K. Maji, Rabindra Bhattacharya, Santanu Guha, Nighat N. Khan, A. Kumar Sinha

**Affiliations:** 1 Sinha Institute of Medical Science and Technology, Garia 700084, India;; 2 Gandhi Memorial Hospital, Kalyani, 741245, West Bengal, India;; 3 Dept. Of Cardiology, Calcutta Medical College and Hospital, Kolkata, India;; 4 RR&D Center of Excellence, James J. Peters VA Medical Center, 130 W. Kingsbridge Road, Bronx, NY, USA;; 5 Sinha Institute of Medical Science & Technology, 288, Kendua main road, Garia, 700 084, India

**Keywords:** Antihypertensive medications, Aspirin, Dermcidin Isoform 2, (r)-Cortexin, Nitric Oxide

## Abstract

Currently, there is no specific medication for essential hypertension (EH), a major form of the condition, in man. As acetyl salicylic acid (aspirin) is reported to stimulate the synthesis of renal (r)-cortexin, an anti-essential hypertensive protein, and, as aspirin is reported to inhibit dermcidin isoform 2 (dermcidin), a causative protein for EH, the role of aspirin in the control of EH in man was studied. Oral administration of 150 mg aspirin/70 kg body weight in subjects with EH was found to reduce both the elevated systolic and diastolic blood pressures to normal levels within 3 h due to the normalization of dermcidin level in these subjects. The plasma cortexin level at day 0, 1, 30 and 90 were 0.5 pmol/ml, 155.5 pmol/ml, 160.2 pmol/ml, 190.5 pmol/ml respectively with increased NO synthesis (r=+0.994). *In vitro* studies demonstrated that the incubation of the goat kidney cortex cells with aspirin stimulated (r)-cortexin synthesis due to NO synthesis. It could be suggested that the use of aspirin might control EH in man.

## INTRODUCTION

Essential hypertension (EH), also known as primary or idiopathic hypertension, is reported to be the major form of hypertension in humans (1). It has been estimated that >90% of all hypertensive subjects are affected by EH (1). Characteristically, the occurrence of EH does not produce any discernable symptoms in the victims, and consequently, many of the subjects affected by EH are not even aware of the existence of the condition in their system. Until very recently there was no laboratory diagnosis of EH (2). And, as such, the treatment of the elevated blood pressure in EH by using various antihypertensive drugs remains a “hit or miss” therapy for the condition, in that, no specific therapy for the elevated blood pressures in EH is currently known. However, it should not be construed that the use of the so called “non specific” antihypertensive medications or their combinations failed to control the elevated blood pressure in EH. Many of these antihypertensive medications however are well known for their unpleasant side effects (3-8). These untoward side effects range from various intestinal problems to sexual dysfunctions particularly in men (7, 8).

We have recently reported the synthesis of an antihypertensive protein (molecular weight 43 kDa), produced in the goat kidney cortex cells, that is trivially called renal (r)-cortexin (9). This antihypertensive protein was found to occur in all mammalian blood including that of humans. Furthermore, it was found that the plasma level of (r)-cortexin in human subjects with EH was reduced to 0 pmol/ml in contrast to >200 pmol/ml in age and sex matched normotensive persons (9). The antihypertensive protein was also found to control the increased systolic blood pressure (SBP) and diastolic blood pressure (DBP) induced by the injection of *l*-epinephrine in the animal model (9).

In the context of the (r)-cortexin as described above, it must be mentioned here that another protein of 82 amino acid residues has been identified before in the cortex of the brain (10), and has also been claimed to be expressed in the kidney cells (10). The brain “cortexin” (82 amino acid residues) protein of ≈ 10,000 Da Mr. cannot possibly be (r)-cortexin, in that, (r)-cortexin had been reported to be of Mr. 43,000 Da (9). More importantly, however, the brain cortexin has no known physiologic function except that the investigators confusingly claimed that this protein “maybe particularly important” for the development of neuron without any evidence whatsoever to that effect, or the involvement of the brain cortexin in the control of elevated blood pressures either in humans or in animals (10, 11). More curiously, how the “cortexin” gene was expressed in the brain cortex cells was not even mentioned by the investigators (11). It should be mentioned here that no gene is automatically expressed to synthesize protein and appropriate stimulator is essential for the synthesis of any gene. In contrast, nitric oxide (NO) was reported to be capable of inducing the actual (r)-cortexin synthesis in the kidney cortex cells both *in vitro* and *in vivo* (9).

Although the development of hypertension in humans, where >90% cases of hypertension has been reported to be EH, has a distinctive feature of being genetically related, neither any gene nor its product (i.e a protein) that could be involved in the genesis of hypertension, has yet been identified. On the other hand, that environmental stresses could lead to hypertension have been repeatedly reported before (12, 13). We, for the first time ever reported the expression of a gene that resulted in the synthesis of dermcidin isoform 2 (dermcidin) in various cells including leucocytes in humans (14, 15). Furthermore, dermcidin was found to be capable of inducing essential hypertension in man (16). We have also reported before that the oral ingestion of acetyl salicylic acid (aspirin) by persons with dermcidin level 112 nM could be reduced to ≈10 nM (considered to be normal) within 24 h due to aspirin induced systemic NO synthesis (14), and not by the classical effect of aspirin through the inhibition of cycloxygenase (17, 18).

As described in the Materials and Methods, a group of “outdoor” patients with undetermined malaise, aches, and pain, who had no knowledge that they were hypertensive, were asked to swallow aspirin for their aches and pain in the body but not any hypertensive medicine for the elevated blood pressures. On follow-up, it was serendipitously found that both the SBP and DBP that were elevated at presentation were reduced to normotensive levels within 3 h.

As the follow-up studies demonstrated that many of these hypertensive subjects actually had EH, the plasma (r)-cortexin level was determined to find out the effect of the aspirin induced (r)-cortexin synthesis in the control of the elevated blood pressures in EH.

The use of aspirin in the context of the impaired (r)-cortexin production in EH was carried out in particular because we have reported before that the generation of NO in the kidney cortex cells resulted in the actual synthesis of (r)-cortexin mRNA in these cells (9). As aspirin has been reported to stimulate NO synthesis (19) through the activation of a constitutive nitric oxide synthase (19), independent of the well known effect of aspirin on the inhibition of cyclooxygenase (17, 18), the effect of aspirin on the inhibition of dermcidin was studied. As dermcidin was reported to be a causative protein for EH in man induced by the environmental stresses, and aspirin has been reported to neutralize the synthesis of dermcidin through NO synthesis, the effects of aspirin on the synthesis of (r)-cortexin and on the inhibition of dermcidin was studied for its possible use as a specific anti-essential hypertensive agent.

## METHODS

### Ethical Clearance

As the study involved human subjects, all participating volunteers were asked to sign an informed consent form. Appropriate permission from the Internal Review Board, Sinha Institute of Medical Science and Technology, Calcutta, was obtained. This study also used kidney from goat obtained from the slaughter house. The permission to use goat kidney for our study was also taken from the IRB.

### Chemicals

The goat anti-rabbit immunoglobulin G-alkaline phosphatase was purchased from Sigma Aldrich. Enzyme–linked immunosorbent assay (ELISA) Maxisorb plates were from Nunc, Roskilde, Denmark. Aspirin was used for the study was obtained from Medica Zydus Healthcare. All other chemicals used were of analytical grade.

### Selection of subjects

The subjects (n=74; M=37, F=37) between the ages of 30-60 years participated in the study. The volunteers who participated in the study were “outdoor patients” in Calcutta Medical College and Hospital, Calcutta. These persons came to the hospital with undefined malaises including aches and pains in the limbs. The cause of these ill-defined aches and pains was unknown to the subjects themselves in most cases.

During the routine check-up, it was found that many of the subjects at presentation had SBP ≥ 140 mm of Hg and DBP ≥ 90 mm of Hg. And, as such, these persons were considered to be hypertensive (1). None of these subjects with hypertension were aware that they were hypertensive and, as such, never received any antihypertensive medications for the condition. The selected volunteers had no diabetes mellitus, or cardiovascular diseases. These selected volunteers had no life threatening infection or had been hospitalized for any condition in the last 6 months. The selected participants had not received any medication or aspirin at least for 14 days. None of the female subjects had ever received any contraceptive medications.

### Administration of aspirin and the rationale for the use of the compound in the subjects

As most of the “outdoor” patients who came to the hospital with undefined “malaise” and non-specific aches and pains, the attending physician opted to ask these persons to swallow 150 mg of aspirin with water, only after these subjects had taken an adequate meal consisting of foods rich in protein like meat (90 gm/70 kg body weight), milk, and cheese and carbohydrate rich foods like bread to “prevent” the possible irritation of the stomach that may be caused by the ingestion of the compound (20). Before the oral administration of aspirin to these subjects as described above, they were randomized and only the willing subjects received aspirin without any regard to the presence or absence of pre existing hypertension. In other words, the investigators had no knowledge of the subjects who were hypertensive or who were not.

The attending physician did not prescribe any antihypertensive drugs because it was thought that the increase of blood pressures in some of the subjects could be due to the “white coat” syndrome and overnight rest might resolve the condition.

The subjects who received aspirin and the subjects who refused to ingest the compound by their own choice were asked to be present the next day for further check- up.

### Preparation of aspirin solution for *in vitro* studies

Fresh acetyl salicylic acid solution was prepared, just before use, by dissolving the compound in 0.1 M NaHCO_3_ and immediately neutralized to pH 7.0 at 0°C which was discarded after use (19).

### Preparation of the goat kidney cortex cells

Fresh kidneys were obtained from the local slaughter house and the cortex cell suspension was prepared in Kreb’s buffer, pH 7.4 as described before (9).

### Synthesis of (r)-cortexin in the goat kidney cortex cells in the presence of aspirin

Typically, 10 mg kidney cortex cells suspension in Kreb’s buffer containing 2.0 nM Ca^2+^ was incubated with different concentrations of aspirin at 37°C in total volume of 2.0 ml for 30 min. The (r)-cortexin synthesis was determined by *in vitro* translation of the (r)-cortexin mRNA using 1 mM ATP and 1 µM each of all 20 amino acids in the reaction mixture by using plant leaves ribosomal particles as described (21). The quantitation of (r)-cortexin synthesis in the incubation mixture was carried out by ELISA as described before (2).

### Statistical analysis

The results shown are mean ± standard deviation (SD). The significance of the results was analyzed by Student’s paired “t” test where difference in the mean values were considered significant with *P*<0.05. One way ANOVA analysis was performed using Newman-Keuls Multiple Comparison Test wherever applicable with a significance of *P*<0.05 using GraphPad Prism. The coefficient of correlation (r) was determined by Pearson test.

## RESULTS

### Effect of aspirin on the elevated blood pressures and dermcidin levels in subjects with essential hypertension

**Figure 1:**
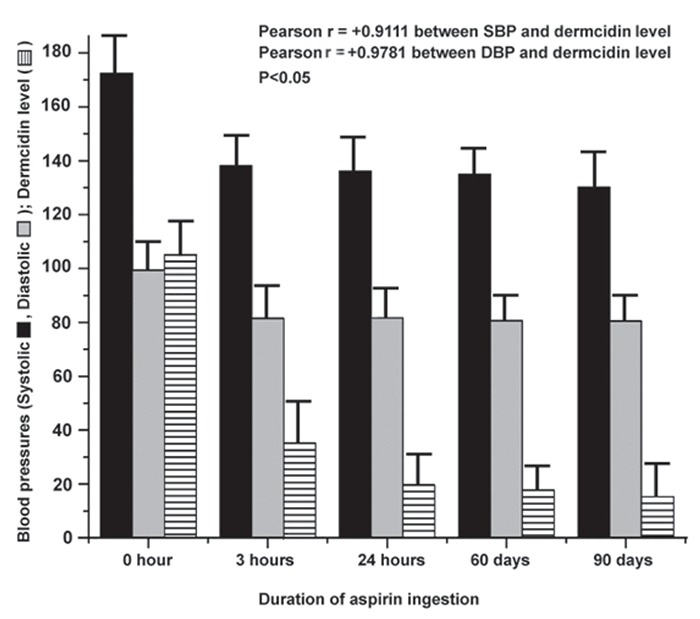
The effect of oral administration of aspirin on the elevated blood pressures after different periods of time. Seventy four hypertensive patients (n=74; M=37, F=37) were administered 150 mg of aspirin orally after an adequate meal as described in the Materials and Methods. Both the SBP and DBP as well as plasma dermcidin levels were measured at different times as indicated. Solid bars (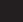) represent the SBP, gray bars (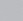) represent the DBP and the patterned bars (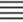) indicate the dermcidin level. The SBP and DBP were measured in each individual at least for 3 times by a mercury sphygmomanometer and expressed in mean ± S.D. The plasma dermcidin levels were measured by ELISA in 3 different experiments, each in triplicate.

As hypertension in humans is considered to be an incurable disease and the condition is kept under control usually by the continuous use of antihypertensive compounds, studies were conducted to determine whether aspirin could be used on a continuing basis to control the elevated blood pressures. For this purpose, a group of subjects who were determined to have EH (n=74; M=37, F=37; between the ages of 30-60 yrs) were randomly selected from the participating subjects as described in the Materials and Methods. These subjects received 150 mg aspirin every 24 h. It was found that the SBP of these subjects which was 172.5 ± 1.6 mm (Mean ± S.D.) of Hg before the administration of aspirin was reduced to 138.2 ± 6.1 mm of Hg at 3 h after the ingestion of the compound. The DBP which was 99.5 ± 2.1 mm of Hg before the use of aspirin was found to be decreased to 81.5 ± 2.9 mm of Hg at the same time (i.e., at 3 h). The continuation of aspirin in these subjects resulted in the decrease of the SBP to 136.2 ± 4.2 mm of Hg at day 30 and to 135 ± 0.5 mm of Hg and 130.2 ± 5 mm of Hg at day 60 and 90 respectively. The DBP at day 30 was 81.7 ± 3.4 mm of Hg, 80.7 mm of Hg at day 60 and 80.5 ± 2.8 mm of Hg at day 90 respectively (Fig. 1). Simultaneously, a group of subjects who had high blood pressures, were not given any aspirin, were treated as control, there was no significant change in SBP (175.5 ± 2.1 mm of Hg), DBP (99 ± 1.9 mm of Hg) and dermcidin level (112.9 nM) during the experiment i.e. up to 90 days.

The plasma dermcidin level was also determined in the same hypertensive subjects before and after the ingestion of the compound at different times as mentioned above. It was found that the dermcidin level which was 105 ± 7.5 nM before aspirin ingestion was decreased to 35.2 ± 2.1 nM after the ingestion of the compound after 3h. Similarly the level of the protein in the plasma of these subjects was found to be reduced to 19.5 ± 2.5 nM, 17.8 ± 1.3 nM and 15.3 ± 1.1 nM on day 30, 60 and 90 respectively (Fig. 1).

The correlation between SBP and dermcidin level as well as DBP and dermcidin level in the experimental group was positively significant (alpha=0.05) with Pearson r value of +0.9111 and +0.9781 respectively. Also one way ANOVA test was performed using Newman-Keuls Multiple Comparison Test which demonstrated that the difference in the mean value of the 3 observations (viz., SBP DBP and dermcidin levels) between the experimental group who opted to ingest aspirin for their condition versus the control group who did not receive aspirin was significant with *P*<0.05.

### Effects of acetyl salicylic acid on the synthesis of (r)-cortexin in the goat kidney cortex cell preparation

We have reported before that the treatment of goat kidney cortex cells preparation with NO solution in 0.9% NaCl resulted in the actual synthesis of (r)-cortexin as evidenced by the *in vitro* translation of (r)-cortexin mRNA and not merely due to the release of the preformed (r)-cortexin from the kidney cortex cells due to aspirin induced NO synthesis. As aspirin has been reported to stimulate NO synthesis in various cells (19), the effect of aspirin on the synthesis of (r)-cortexin through NO synthesis in goat kidney cortex cells preparation *in vitro* was studied as described in the Materials and Methods.

**Figure 2:**
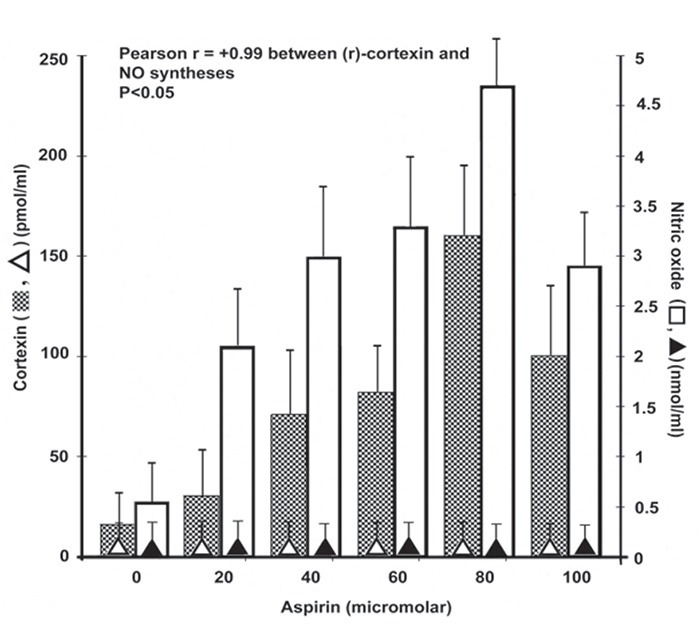
Effect of different concentrations of aspirin on the syntheses of (r)-cortexin and NO in goat kidney cortex cells preparation. Kidney cortex cells were prepared from goat kidney as described in the Materials and methods. Typically 1mg of cortex cells suspended/ml of Tyrod’s buffer was treated with different amounts of aspirin as indicated. After 30 mins of incubation at 37°C, the syntheses of (r)-cortexin and NO were determined. Hollow bars (□) and solid triangles (▲) represent the NO synthesis in the absence and presence of L-NAME respectively; the dotted bars (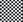) and the hollow triangles (Δ) represent the (r)-cortexin production in the absence and presence of L-NAME respectively. Each experiment was carried out at least in 5 different kidney cells preparation using different kidney cortex and expressed in Mean ± S.D.

It was found that the incubation of goat kidney cortex cells preparation with different concentrations of aspirin for 30 minutes at 37°C (optimal time) resulted in the increased syntheses of both (r)-cortexin and NO (*P*<0.05). When the aspirin induced synthesis of NO in the kidney cortex cells was simultaneously determined, it was found that the increase of NO synthesis was highly correlated to the amount of (r)-cortexin synthesis [Coefficient of correlation between synthesis of NO and (r)-cortexin was +0.99] (Fig. 2). The inhibition of aspirin induced NO synthesis by the addition of 0.1 mM NAME an inhibitor of acetyl salicylic acid induced NO synthesis (18) resulted in the inhibition of both NO and (r)-cortexin synthesis (*P*<0.05). The addition of 0.1 mM NO solution in 0.9% NaCl to kidney cortex cells preparation instead of aspirin was also found to stimulate the (r)-cortexin synthesis *in vitro* from the basal 35.1 ± 3.1 pmol/ml to 52 ± 1.5 pmol/ml at the same time.

### Effects of different antihypertensive compounds and aspirin on the synthesis of NO and (r)-cortexin in the kidney cortex cell preparation

As described above, oral administration of aspirin in hypertensive persons resulted in the reduction of both SBP and DBP that were related to the increase of the plasma (r)-cortexin level. In an effort to determine the effects of different antihypertensive medications that are commonly used in the treatment of hypertension, the synthesis of NO and (r)-cortexin in the goat kidney cortex cells preparation were incubated with these compounds and the syntheses of both these components were determined. As described in the Table 1, the addition of different antihypertensive drugs to the *in vitro* reaction mixture at therapeutic doses when added to the reaction mixture were capable of stimulating both NO and (r)-cortexin syntheses. These results suggested that the different antihypertensive compounds, like aspirin itself, were capable of stimulating (r)-cortexin synthesis due to NO synthesis. However aspirin was found to be more efficient in the synthesis of (r)-cortexin when compared to the other antihypertensive compounds used in the study.

**Table 1:** The effect of different antihypertensive medications on the syntheses of (r)-Cortexin and NO in goat kidney cortex cells *in vitro*

Name of anti-hypertensive medications	Level of cortexin (pmol/ml)	aStudent’s paired “t” test “p” value of cortexin level	Level of NO (nmol/ml/hr)	^*^Student’s paired “t” test “p” value of NO level	“r” value between cortexin and NO

No medication	32.6 ± 3.15	----	2.650 ± 0.123	-----	+0.900
Calcium channel blocker (Nifedipine) (0.017 μmol/ml)	156.94 ± 5.26	<0.05	2.818 ± 0.245	<0.05	+0.847
Calcium channel blocker (Amloguard) (0.001 μmol/ml)	152.74 ± 5.28	<0.05	4.404 ± 0.421	<0.05	+0.980
Spironolactone (Aldactone) (0.03μmol/ml)	138.16 ± 6.21	<0.05	3.66 ± 0.321	<0.05	+0.999
Aspirin (0.08 μmol/ml)	160.67 ± 7.19	<0.05	5.644 ± 0.512	<0.05	+0.999
Aspirin (0.1 μmol/ml)	145.25 ± 3.52	<0.05	3.458 ± 0.216	<0.05	+0.990
bL-NAME (0.1 μmol/ml)	0	<0.05	0.040 ± 0.002	<0.05	+0.970

a Student’s paired “t” test “p” value in case of each antihypertensive medication as well as aspirin was calculated against the control experiment in the absence of any medication with a 95% confidence interval.

b L-NAME is not an anti-hypertensive agent but it is used as an inhibitor of NO. Its inclusion in the study was only to indicate the inhibition of NO synthesis that in turn led to the inhibition of cortexin synthesis in the kidney cortex cells. Goat kidney cortex cells suspension was prepared in Tyrod’s buffer, pH7.4, as described in the Materials and Methods. Typically 1mg of cell suspension/ml of Tyrod’s buffer was treated with different anti- hypertensive medications as indicated. After incubation for 30 min at 37°C, syntheses of (r)-cortexin and NO were measured. The amounts of medications used are shown in parentheses under the name of each compound. For convenience, the trade name of each compound is also shown within the parenthesis. The results shown are Mean ± S.D. of 5 different experiments using 5 different kidney cortex cells preparation.

A one way ANOVA analysis was performed which demonstrated that the variances between the control group (i.e., cortexin and NO synthesis in the absence of aspirin) v/s the experimental group (i.e., cortexin and NO synthesis in the presence of aspirin) differed significantly with *P*<0.05. Additionally Newman-Keuls Multiple Comparison Test suggested that the means between the groups were also significantly different with *P*<0.05.

### Effect of aspirin on the reduction of systolic and diastolic blood pressures in subjects with EH and the systemic increase of (r)-cortexin level

We have reported before that the plasma (r)-cortexin level in subjects with EH was reduced to 0 pmol/ml (9). To find out the effect of the oral ingestion of 150 mg of aspirin in subjects with EH, a group of volunteers (n=74; M=37, F=37, between the ages of 30-60 years) were selected from the “outdoor” subjects who were determined to have EH and were randomly selected as described in the Material and Methods. These subjects with EH were asked to swallow 150 mg of aspirin with water only after they had taken an adequate meal as described (22). Prior to swallowing aspirin, 10 ml of blood was drawn and anti-coagulated by using 0.13 mM sodium citrate as described before (23).

**Figure 3:**
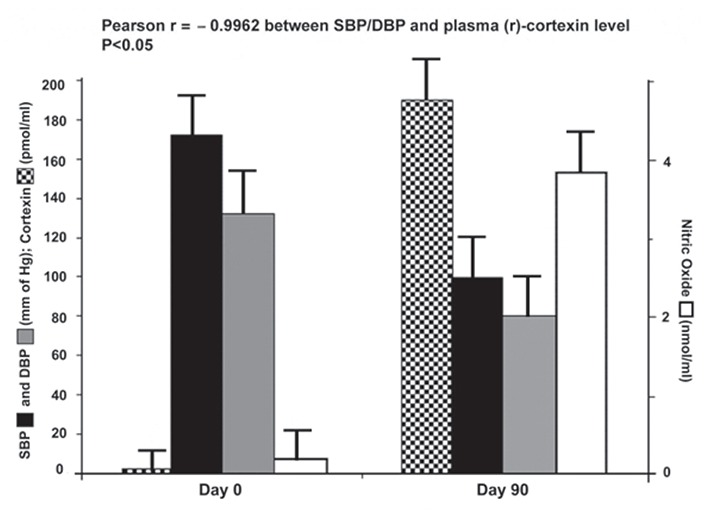
Effect of ingestion of aspirin by hypertensive subjects for different times on Systolic Blood Pressure (SBP), Diastolic Blood Pressure (DBP), (r)-cortexin and NO levels. Hypertensive subjects (n=74; M=37, F=37) were asked to ingest 150 mg of aspirin as described in the Materials and Methods. These subjects continued aspirin ingestion every 24 h for 3 months. Both SBP and DBP were measured before and after the ingestion of the compound and the plasma NO and cortexin levels were also determined in these subjects. Solid bars (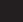) represent the Systolic Blood Pressure (SBP). Gray bars (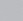) represent the Diastolic Blood Pressure (DBP). Hollow bars (□) indicate the NO synthesis. Dotted bars (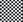) represent the (r)-cortexin synthesis. The results are mean ± S.D. of the participants (n=74) (*p*<0.05).

An equal number of age and gender matched subjects with EH who at their own wish did not receive aspirin, but had taken an adequate meal served as the controls. Both SBP and DBP were determined after 3 h in both these groups of subjects with or without the ingestion of aspirin and blood samples were again collected after 3 h from these subjects. The plasma cortexin level was also determined which was found to be increased from 0.5 pmol/ml to 110 pmol/ml in those subjects who has ingested aspirin. The experiment was extended for 90 days and SBP and DBP were determined along with the assay of plasma cortexin levels. After 24 h of ingestion of aspirin, the plasma cortexin level was 155.5 pmol/ml, and after 30 days the level of cortexin was found to be 160.2 pmol/ml with a reduction of both SBP and DBP to 135 ± 0.5 mm of Hg and 81.7 ± 3.4 mm of Hg respectively. The cortexin level after 90 days was 190.5 pmol/ml with increased NO synthesis (r=+0.994) (Fig. 3).

On the contrary, the control group consisting of the subjects who did not receive aspirin, had their plasma (r)-cortexin level at ranges of <0.5 nM with increased SBP, DBP and dermcidin levels (112.9 nM).

The subjects were randomized before the SBP and DBP were measured and the plasma (r)-cortexin levels were determined (Fig. 3) in a “double-blind” manner, in that, the investigators had no knowledge about the subjects who had or had not ingested aspirin. The data were tabulated by a third person who was not involved in the study.

The correlation between the SBP/DBP and cortexin level in those hypertensive subjects who had ingested aspirin was determined to be negative with Pearson r value of -0.9962. The difference in the variances and the means as determined by Newman-Keuls Multiple Comparison Test between the different observations in the control and experimental groups was found to be significant with P<0.05.

## DISCUSSION

These results suggested that aspirin might be used as an antihypertensive compound. It was found that the aspirin induced stimulation of NO synthesis that consequently stimulated the (r)-cortexin synthesis in the kidney cortex cells resulted in the reduction of both SBP and DBP in the essential hypertension in man. This inference was based on our earlier report that the plasma (r)-cortexin in EH in humans was found to be reduced to 0 pmol/ml that contrasted >200 pmol (r)-cortexin/ml in normotensive persons (9). It could be argued that as aspirin was capable of stimulating systemic NO synthesis (19) which itself has been reported to be a global vasodilating compound (24), what could be the physiologic advantage of a multistep energy requiring process at the expense of ATP/GTP for the synthesis of (r)-cortexin, (a protein) in the kidney cortex cells (9). However, the half-life of aspirin induced systemic production of NO is extremely small (≈10^-8^sec) in the presence of O_2_ in the circulation and, as such, NO may not be available to reach the vascular system to control the elevated blood pressures. On the other hand, (r)-cortexin, a protein hormone (43,000 Mr.) has been reported to activate a membrane nitric oxide synthase (NOS) in the endothelial cells, which themselves have no basal NOS activity, and the presence of (r)-cortexin activated nitric oxide synthase in the endothelial cells for the control of the elevated blood pressure through the synthesis of NO (9).

As described in the Table 1, different antihypertensive medications, including different calcium ion channel blockers, β adrenergic blocker, spironolactone, all were capable of stimulating (r)-cortexin synthesis in the kidney cortex cells preparation *in vitro* with simultaneous stimulation of NO synthesis *in vitro*. Although the constitutive form of NOS that is activated by aspirin has been purified to homogeneity and the characteristics of the enzyme has been studied in details (19), the mechanism of NO synthesis *in vitro* by the different antihypertensive compounds (Table 1) is not known currently. However, except in the case of aspirin, the oral administration of the above antihypertensive drugs at their therapeutically effective doses failed to increase the plasma (r)-cortexin level (2). The mechanisms for the failure of the antihypertensive compounds at recommended therapeutic dose to increase the plasma (r)-cortexin level remain obscure. It is possible that the therapeutic dose of these compounds was not sufficiently high for the increase of (r)-cortexin levels *in vivo*. On the other hand, the site of (r)-cortexin production was reported to be the kidney cortex which is actually an afferent vascular system and serve as the exit point for many compounds present in the circulation, as such, it was possible that the above mentioned antihypertensive compounds did not get optimal time for the interaction with the kidney cortex cells for the synthesis of (r)-cortexin through NO synthesis. However, other mechanisms are also possible. In contrast the use of ^14^C aspirin was found to bind to cNOS in different cells to stimulate NO synthesis (unpublished).

Although the mechanisms involved in the reduction of the elevated blood pressures by the above mentioned antihypertensive compounds have already been reported (25, 26), it is still possible that the increase of (r)-cortexin synthesis by these compounds might have a contributory role to control EH. This inference was made due to the fact that the vast majority of the hypertensive subjects are reported to have EH, and the elevated blood pressures in these subjects are controlled by using “non-specific” antihypertensive compounds (Table 1), which implied thereby that the elevated blood pressures in EH could be controlled, at least in some cases through the synthesis of (r)-cortexin. Furthermore, it might be possible that EH could be controlled by the “staggering” effect of some of these antihypertensive compounds due to their continuous use.

In the above context, it should also be mentioned here that several unrelated environmentally induced stresses are reported to induce hypertension including the essential form of the condition in humans, due to the systemic expression of the genes in different cells including leucocytes in the circulation that led to the synthesis of dermcidin isoform 2 (15), which probably is the only protein induced by environmental stresses currently known to induce hypertension in the animal model and was found to cause even EH in humans (16). We have reported before (16), and as it has also been described in the present study (Fig. 1 and Fig. 3) that the oral administration of aspirin was capable of reducing both the elevated SBP and DBP through the neutralization of the systemic dermcidin level to normal ranges in subjects affected by EH.

These results as described from our study suggested that it might be possible to conclude that aspirin could be a specific antihypertensive compound for EH not only through the synthesis of (r)-cortexin but also through the neutralization of systemic dermcidin level in EH. Thus aspirin was capable of controlling EH in humans through two unrelated and independent mechanisms both of which are reported to be involved in control of blood pressures in EH (9, 16). It should also be mentioned here that although the effect of aspirin in the reduction of hypertension in general but not in EH, has been reported before (27-29), neither the mechanism of aspirin induced reduction of hypertension nor the involvement of any mediators for the control of elevated blood pressures in these studies were even mentioned.

The use of aspirin has been reported before to reduce the occurrence of acute ischemic heart disease (AIHD) through the inhibition of prostaglandins syntheses (30). However, AIHD itself has been reported to be a consequence of atherosclerosis (31). Both diabetes mellitus and hypertension are the two known major risk factors for atherosclerosis (32). As aspirin has been reported before to be an anti-diabetic agent (33), and as the compound is also described here to be an antihypertensive agent, aspirin might be the “compound of choice” to prevent atherosclerosis leading to AIHD, in that, none of the commonly used antihypertensive drugs (Table 1) unlike aspirin itself, was capable of stimulating insulin synthesis in the presence of glucose and NO which was generated by aspirin due to the activation of cNOS (19). The effect of aspirin in these cases was not mediated through the well known classical effect of aspirin on the inhibition of cyclooxygenase but through the activation of nitric oxide synthase (19).

Higher doses of aspirin is well known to produce prostaglandin in intestinal wall and may cause ulcer because of the lack of development of intestinal mucosa and the persons who are suffering from duodenal ulcer will not be advised to take aspirin as an antihypertensive medication. Far rarer cases, the persons who have allergy to aspirin, should not take aspirin. In these cases aspirin should not be taken to avoid some untoward effects of the compound. Furthermore, it is not a placebo controlled study. The persons who refused to take aspirin for their own personal reasons despite the fact hypertension were not the placebo control. Ambulatory blood pressure monitoring (ABPM) is also not used here because it has some limitations. It is mainly used in case of “white coat hypertension”. Clinic hypertension measurement machine is widely used in medical hospitals by the doctors and nurses. The effect of antihypertensive medications is measured by clinic hypertensive measurement machine and the drawback of ABPM is that it cannot provide correct result of arrythmia, elderly persons, and high blood pressure patients. Some background noise also interfere with the ABPM readings and there is also interpretable problems of ABPM readings. Above all, it is controversial till now as to which is better ABPM or clinic hypertensive measurement machine.

Finally it should also be mentioned that many of the untoward effects including sexual dysfunctioning and serious intestinal problems in the users produced by many commonly used antihypertensive drugs as described above (Table 1) are usually not produced by aspirin at the dose which is described in Table 1. And, as such, aspirin could also be a more useful antihypertensive medication for EH in that context.
